# In vivo probabilistic atlas of white matter tracts of the human subthalamic area combining track density imaging and optimized diffusion tractography

**DOI:** 10.1007/s00429-022-02561-3

**Published:** 2022-09-17

**Authors:** Gianpaolo Antonio Basile, Marina Quartu, Salvatore Bertino, Maria Pina Serra, Marcello Trucas, Marianna Boi, Roberto Demontis, Alessia Bramanti, Giuseppe Pio Anastasi, Demetrio Milardi, Rosella Ciurleo, Alberto Cacciola

**Affiliations:** 1 Brain Mapping Lab, Department of Biomedical, Dental Sciences and Morphological and Functional Images, University of Messina, Messina, Italy; 2 Department of Biomedical Sciences, Section of Cytomorphology, University of Cagliari, Cittadella Universitaria di Monserrato, 09042 Monserrato (CA), Italy; 3 Department of Medical Sciences and Public Health, University of Cagliari, 09042 Monserrato (CA), Italy; 4 Department of Medicine, Surgery and Dentistry “Medical School of Salerno”, University of Salerno, Salerno, Italy; 5 IRCCS Centro Neurolesi “Bonino Pulejo”, Messina, Italy

**Keywords:** Anatomy, Basal ganglia, Brain, Cerebellum, Thalamus

## Abstract

The human subthalamic area is a region of high anatomical complexity, tightly packed with tiny fiber bundles. Some of them, including the pallidothalamic, cerebello-thalamic, and mammillothalamic tracts, are relevant targets in functional neurosurgery for various brain diseases. Diffusion-weighted imaging-based tractography has been suggested as a useful tool to map white matter pathways in the human brain in vivo and non-invasively, though the reconstruction of these specific fiber bundles is challenging due to their small dimensions and complex anatomy. To the best of our knowledge, a population-based, in vivo probabilistic atlas of subthalamic white matter tracts is still missing. In the present work, we devised an optimized tractography protocol for reproducible reconstruction of the tracts of subthalamic area in a large data sample from the Human Connectome Project repository. First, we leveraged the super-resolution properties and high anatomical detail provided by short tracks track-density imaging (stTDI) to identify the white matter bundles of the subthalamic area on a group-level template. Tracts identification on the stTDI template was also aided by visualization of histological sections of human specimens. Then, we employed this anatomical information to drive tractography at the subject-level, optimizing tracking parameters to maximize between-subject and within-subject similarities as well as anatomical accuracy. Finally, we gathered subject level tracts reconstructed with optimized tractography into a large-scale, normative population atlas. We suggest that this atlas could be useful in both clinical anatomy and functional neurosurgery settings, to improve our understanding of the complex morphology of this important brain region.

## Introduction

The human subthalamic area is an anatomically complex region of the diencephalon containing nuclear groups and white matter fascicles tightly packed together (Nieuwenhuys 2008). Many of the white matter structures crossing this region convey major input to specific thalamic nuclei (Gallay et al. 2008). Such fiber pathways include the ansa lenticularis (AL) and the fasciculus lenticularis (FL), which connect the internal globus pallidus (GPi) to the ventral anterior nucleus of the thalamus (VA); the cerebello-thalamic tract (CTT), which emerges from the dentate nucleus of the cerebellum and travels through the superior cerebellar peduncle to reach the posterior subdivision of the ventral lateral nucleus (VLp), mostly in its inferior aspect (ventral intermediate nucleus, Vim, using Hassler’s nomenclature) (Hassler 1959; Hirai and Jones 1989; Mai and Majtanik 2019); the mammillothalamic tract (MTT), which connects the anteromedial mammillary body to the anterior nuclear group of the thalamus (Ant); and the medial lemniscus (MedL), which connects sensory relays of the brainstem to the ventral posterior (VP) nucleus (Neudorfer and Maarouf 2018; Middlebrooks et al. 2020).

White matter tracts of the subthalamic area have gained increasing attention as a potential target in stereotactic neurosurgery. Once crucial for surgical lesion approaches such as subthalamotomy (Velasco et al. 1972, 1975) or pallidothalamic tractotomy (Aufenberg et al. 2005), to date, white matter ablation has been largely replaced by deep brain stimulation (DBS) of gray matter structures: in particular, the subthalamic nucleus (STN) represents one of the main targets for the treatment of Parkinson’s disease (PD) (Aviles-Olmos et al. 2014), the Vim for essential tremor (ET) and tremor-dominant PD (Flora et al. 2010; Cury et al. 2017), the GPi for dystonia and PD-related dyskinesias (Taira 2015; Liu et al. 2019), and the Ant for treatment resistant epilepsy (Möttönen et al. 2015). However, recent evidence from both clinical and neuroimaging studies has suggested that better clinical outcomes may be obtained when the stimulation is delivered in proximity to specific white matter tracts, such as the AL/FL for dyskinesia (Aquino et al. 2019), the CTT for tremor (Al-Fatly et al. 2019), or the MTT tract for epilepsy (Schaper et al. 2020). These findings have led to a renewed interest in disentangling the complex white matter anatomy of the subthalamic area (Petersen et al. 2019; Chung and Park 2020; Lau et al. 2020).

In the last decade, diffusion-based tractography has been widely employed to reconstruct white matter bundles in vivo (Jeurissen et al. 2019). Nonetheless, tractography of fiber pathways is significantly influenced by a number of technical factors such as the choice of tracking algorithm and specific tracking parameters (Maffei et al. 2019; Bertino et al. 2021). In addition, tractography can be affected by false-positive streamlines, which decrease its anatomical accuracy (Maier-Hein et al. 2017). Considering that diencephalic tracts are characterized by complex course, these issues are further complicated by the inherent limitations of tractography in resolving complex fiber trajectories, as well as by the low resolution of diffusion MRI acquisition, which impairs the visibility of such tracts and the selection of “waypoints” to guide the virtual dissection. For these reasons, the reconstruction of diencephalic white matter tracts has been performed sporadically, and generally not on a single-subject basis, but rather relying on ex vivo imaging (Plantinga et al. 2016; Chung and Park 2020; Oishi et al. 2020) or on group-averaged estimates of in vivo brain connectivity, such as normative connectomes (Petersen et al. 2019; Middlebrooks et al. 2020). To the best of our knowledge, a population-based, in vivo probabilistic atlas of subthalamic white matter tracts is still missing. On the other hand, building such an atlas would require the solution of non-negligible technical issues, such as (i) the choice of adequate tracking algorithms and parameters to ensure maximal stability of the results across subjects, (ii) the adequate in vivo visualization of white matter structures to allow for identification of false positive tracts, and (iii) a sufficiently large population with high-quality diffusion data.

Herein, we aimed at building an optimized, probabilistic population atlas of white matter tracts of the subthalamic area, by employing 3T high-quality diffusion data from the human connectome project (HCP) repository. We employed track-density imaging (TDI) (Calamante et al. 2010, 2012b) to improve the visualization of white matter structures on a super-resolution TDI population template (Basile et al. 2021a). By visualizing histological sections of human specimens and leveraging the high anatomical details provided by short tracks TDI (stTDI), we were able to follow the entire course of white matter tracts through the subthalamic region and to identify suitable waypoints that were used to drive fiber tracking at the subject level. To select, for each tract, a “best working” and reliable pipeline, tractography was optimized by testing two different tracking algorithms (deterministic vs probabilistic) with different combinations of tracking parameters. Each tractography pipeline was evaluated in terms of both within-subject and between-subject similarity, as well as of similarity with manually delineated tracts on the high-resolution stTDI population template. Finally, the optimized probabilistic atlas has been made available in a commonly employed stereotactic space (Fonov et al. 2009).

## Materials and methods

### Subjects and data acquisition

Minimally preprocessed structural and diffusion MRI data have been obtained from the HCP repository. Two datasets have been employed for the present work: the first dataset (main dataset) consisted of 210 healthy subjects (males = 92; females = 118; age range 22–36 years) and the second dataset (test–retest dataset) included 44 subjects with available test–retest MRI scans (males = 13; females = 31; age range 22–36 years). Notice that 11 subjects of the main dataset were also part of the test–retest dataset. Data were acquired by the Washington University, University of Minnesota, and Oxford University (WU‐Minn) HCP Consortium (Van Essen et al. 2013). The Washington University Institutional Review Board (IRB) approved all the study procedures, including subject recruitment, informed consent and sharing of deidentified subject data. MRI scans were acquired using a Siemens 3T Skyra scanner (Siemens Healthcare, Erlangen, Germany) customized with a Siemens SC72 gradient coil and stronger gradient power supply with maximum gradient amplitude of 100 mT/m with the aim of improving diffusion imaging (Van Essen et al. 2012). Structural scans included T1‐weighted images, acquired with the following parameters: TE = 2.14 ms, TR = 2,400 ms, and voxel size = 0.7 mm (Uǧurbil et al. 2013). A single‐shot two‐dimensional (2D) spin‐echo multiband echo planar imaging (EPI) sequence was used to acquire multi-shell diffusion weighted images (DWI) (b values of 1000, 2000, and 3000 s/mm2), with isotropic spatial resolution of 1.25 mm (Sotiropoulos et al. 2013). Data were available in a minimally preprocessed form that includes correction of EPI susceptibility, eddy‐current-induced distortions, gradient nonlinearities, subject motion, and within‐subject co‐registration of structural and diffusion images (Glasser et al. 2013).

### FOD template estimation and super-resolution track-density imaging

Group level, super-resolved short-tracks track density images (stTDI) were obtained from DWI data of a subsample of 100 unrelated subjects (males = 46; females = 54; age-range 22–36 years) of the main dataset. The whole procedure of DWI post-processing, tractography and stTDI generation is extensively described elsewhere (Basile et al. 2021a). In brief, diffusion signal modeling was performed by applying multi-shell, multi-tissue constrained spherical deconvolution (MSMT-CSD), an optimized version of the CSD signal modeling, which estimates white matter Fiber Orientation Distribution (FOD) function from the diffusion-weighted deconvolution signal using a single fiber response function (RF) as reference (Tournier et al. 2008, 2012). MSMT-CSD improves the classical CSD approach calculating different response functions for gray matter, white matter and cerebrospinal fluid (Tournier et al. 2008; Jeurissen et al. 2014). An unbiased FOD template was built from individual FOD datasets using a dedicated FOD registration algorithm that includes FOD reorientation using apodized delta functions (Raffelt et al. 2011, 2012; Pietsch et al. 2017); the resulting template was cropped to the size of a manually drawn bounding box covering the entire diencephalon. This bounding box was drawn on an axial section at the level of the anterior commissure (z = 0), considering the edge of the putamen as the anterior boundary and extending posteriorly to include the temporal horn of lateral ventricles, bilaterally; lateral boundaries were tangent to the most lateral edge of the putamen; this rectangular ROI was extended on each axial section until reaching the last slice in which the body of the caudate nucleus was visible as the superior boundary, and the section at the level of the optic chiasm as the inferior boundary. 50 million short tracks were seeded from the FOD template within the bounding box (maximum track length = 10 mm; minimum track length = 5 mm; cutoff: 0.05; step size: 0.25 mm). Tracts were mapped to obtain two super-resolution (0.25 mm^3^ voxel size) maps (stTDI): one with directionally-encoded color contrast (DEC), the other with an apparent fiber density-weighted (AFD) contrast, i.e. by assigning to each streamline a weighting that is proportional to the mean FOD amplitude along the streamline itself (Calamante et al. 2012b).

### Histological analysis of human specimens

Parallel post-mortem histological analysis was carried out on specimens (Table 1), with no signs of neuropathology, of the brainstem at the level of the mesencephalic–diencephalic junction and of the whole brain. Specimens of cases 1–6 were fixed by immersion in 4% freshly prepared phosphate-buffered formaldehyde, pH 7.3, for 48 h at 4 °C. As for cases 7 and 8, the extracted brains underwent a first immersion fixation in the same fixative solution for 72 h at 4 °C, and then were dissected to obtain a horizontal brain slice, about 2.5 cm thick, having as inferior boundary the oblique plane passing between the superior surface of the optic chiasm and the dorsal surface of the cerebellum. The horizontal slice was post-fixed in the same freshly prepared fixative solution for further 72 h at 4 °C. All fixed specimens were then rinsed for 24 h in 0.1 M phosphate buffer, pH 7.3, containing 20% sucrose. A rectangular parallelepiped brain tissue block, obtained at approximately the same diencephalic level of the bounding box used for the super-resolution track-density imaging, was then cropped from the brain slice. Specimens were cut with a cryostat. The 16-μm-thick sections were collected in a series of adjacent slices on chrome alum–gelatin-coated slides, and then processed with Klüver and Barrera method for myelin staining. Slides were observed with an Olympus BX61 microscope and digital images were acquired with a Leica DFC450 C camera. Panoramic image photomontages were obtained with the Microsoft Image Composite Editor software (https://www.microsoft.com/en-us/research/product/computational-photography-applications/image-composite-editor/).

**Table 1:** List of specimens

Case	Age	Gender	Causa mortis	Post-mortem interval	Specimen
1	41 y	M	Cardiorespiratory failure	56 h	Mesencephalic–diencephalic junction
2	43 y	F	Fall from height	50 h	
3	44 y	M	Stabbing	50 h	
4	46 y	M	Cardiac tamponade	50.5 h	
5	51 y	M	Myocardial infarction	93 h	
6	65 y	F	Pointed and edged weapon	72 h	
7	60 y	F	Gunshot wound	45 h	Whole brain
8	79 y	M	Gunshot wound	24 h	

*F* female, *h* hours, *M* male, *y* years

### Tracts identification and regions of interest (ROIs) delineation

White matter tracts of interest were identified bilaterally in their entire mesodiencephalic course by an expert neuroanatomist (D.M.). Tracts of interest included the ansa lenticularis (AL), the cerebello-thalamic tract (CTT), the fasciculus lenticularis (FL), the mammillothalamic tract (MTT) and the medial lemniscus (MedL). Tracts were labeled in axial slices on the template-level DEC-stTDI map, with the AFD-stTDI map as overlay (~ 80% opacity). Tracts of interest were identified: (i) according to their anatomical course, as described in the literature and from previously existing imaging and post-mortem dissection papers (Nauta and Mehler 1966; Gallay et al. 2008; Haber 2016; Chung and Park 2020; Holanda et al. 2020; Middlebrooks et al. 2020); (ii) by direct comparison between stTDI slices and diagrams from a well-known stereotactic anatomical atlas (Morel et al. 1997); (iii) by directly comparing stTDI slices with histological slices from human specimens (see previous section); in particular, after careful examination of all specimens, one adult female of 60 years (Case 7) was chosen for tract identification and imaging comparison. Criteria for choice were the quality of sections, tissue preservation, and completeness of the section series. Three single-slice manually delineated waypoint ROIs were placed along the course of each tract bilaterally; additionally, an exclusion waypoint mask covering the internal capsule was also drawn. A manual segmentation of the whole course of each tract was also performed bilaterally on the high-resolution stTDI maps by the trained neuroanatomist and was employed as reference for further investigations (see below).

### Subject-level tractography

Group-level waypoint ROIs for each tract were adapted to guide tractography at the subject-level as follows: first, ROIs were expanded to cover two additional slices upwards and two additional slices downwards (total: 5 slices, i.e., 1.25 mm thickness) to ensure optimal resampling to the lower resolution subject-level diffusion space. Inverse transformations between individual FOD maps and FOD template were obtained using ANTs symmetric diffeomorphic non-linear registration tool (SyN) both for the main dataset (210 subjects) and the test–retest dataset (44 test + 44 retest) (Avants et al. 2008) as detailed in a previous work (Basile et al. 2021a); in particular, individual FOD maps were coregistered to the FOD template using a generic affine transformation by concatenating center-of-mass alignment, rigid, similarity, and fully affine transformations (similarity metric: mutual information), followed by a nonlinear transformation (symmetric diffeomorphic normalization transformation model with regular sampling, similarity metric: cross correlation, gradient step size: 0.2, four multiresolution levels, smoothing sigmas: 3, 2, 1, 0 voxels [fixed image space], shrink factors: 8, 4, 2, 1 voxels [fixed image space], data winsorization [quantiles: 0.005, 0.995], convergence criterion: slope of the normalized energy profile over the past 10 iterations < 10^−6^) that were concatenated into a single composite warp field. The inverse transformations were applied to transform each waypoint ROI, the exclusion mask, and the diencephalic bounding box ROI to individual diffusion space. DWI images were cropped to the bounding box ROI limits and upsampled to 1 mm^3^ isotropic voxel size using cubic interpolation (Dyrby et al. 2014); FODs were estimated from cropped, upsampled DWI data using unsupervised, multi-tissue response functions obtained from DWI only (Dhollander et al. 2016, 2019). Finally, tractography was performed on the resulting FODs using a seed-based approach by selecting, for each tract, 250 streamlines seeded from each waypoint ROI, setting the other waypoint ROIs as the inclusion mask and the waypoint ROIs of other tracts and internal capsule as the exclusion masks. A final tractogram was obtained for each tract by concatenating the tractograms obtained from each of the three waypoint ROIs. All the DWI processing and tractography steps were performed with the MRtrix3 software (Tournier et al. 2019).

### Tractography optimization and atlas generation

To ensure robustness of the obtained tractography results across all subjects, tractography was optimized by iterating tractogram generation with different combinations of tractography algorithms (deterministic, probabilistic) and tracking parameters (step size and angle threshold). In detail, IFod2 (Tournier et al. 2010) was chosen as the probabilistic algorithm and SD_STREAM (Descoteaux et al. 2009) as the deterministic algorithm; the tracking parameters included default (step size 0.5 and angle threshold 45° for probabilistic; step size 0.1 and angle threshold 60 for deterministic), and all the possible combinations between step sizes in the range [0.25, 0.5, 1, 1.25] and angle thresholds in the range [20°, 30°, 60°, 80°], resulting in a total of 34 combinations. After tractography, each tractogram was converted to a TDI tract map and, with a threshold at 15% of its streamline density, binarized and transformed to the FOD group template space using the nonlinear transformations previously described. Optimization was based on three outcome measures: within-subject similarity, between-subject similarity, and anatomical accuracy. All these outcome measures were computed for each combination of parameters and each tract separately using binarized tract maps in template space. Anatomical accuracy was based on the similarity between individual TDI tract maps reconstructed in the main dataset (*n* = 210) to manually segmented reference tracts and it was defined, for each tract map, as the ratio between the number of voxels included into the manually delineated mask (i.e., assumed as belonging to “true positive” tracts) over the number of voxels of the tract map itself. The outcome measures for between-subjects and within-subject similarity are based on the Dice similarity coefficient (DSC) (Dice 1945), defined as

$$DSC=\frac{2 |A\cap B|}{\left|A\right|+|B|}$$

being A and B expressed as numbers of voxels in two images. Specifically, within-subject similarity was calculated from the test–retest dataset and defined as the subject average of DSC of binarized tract maps of the same tract in test (*n* = 44) and retest (*n* = 44) data of each subject; between-subject similarity was calculated from the main dataset (*n* = 210) and defined as the DSC of binarized tract maps of the same tract averaged across each possible pairwise combination of subjects. All the selected outcome measures provide as a result a value ranging between 0 and 1 where 0 means no similarity at all and 1 means absolute identity.

**Fig. 1:**
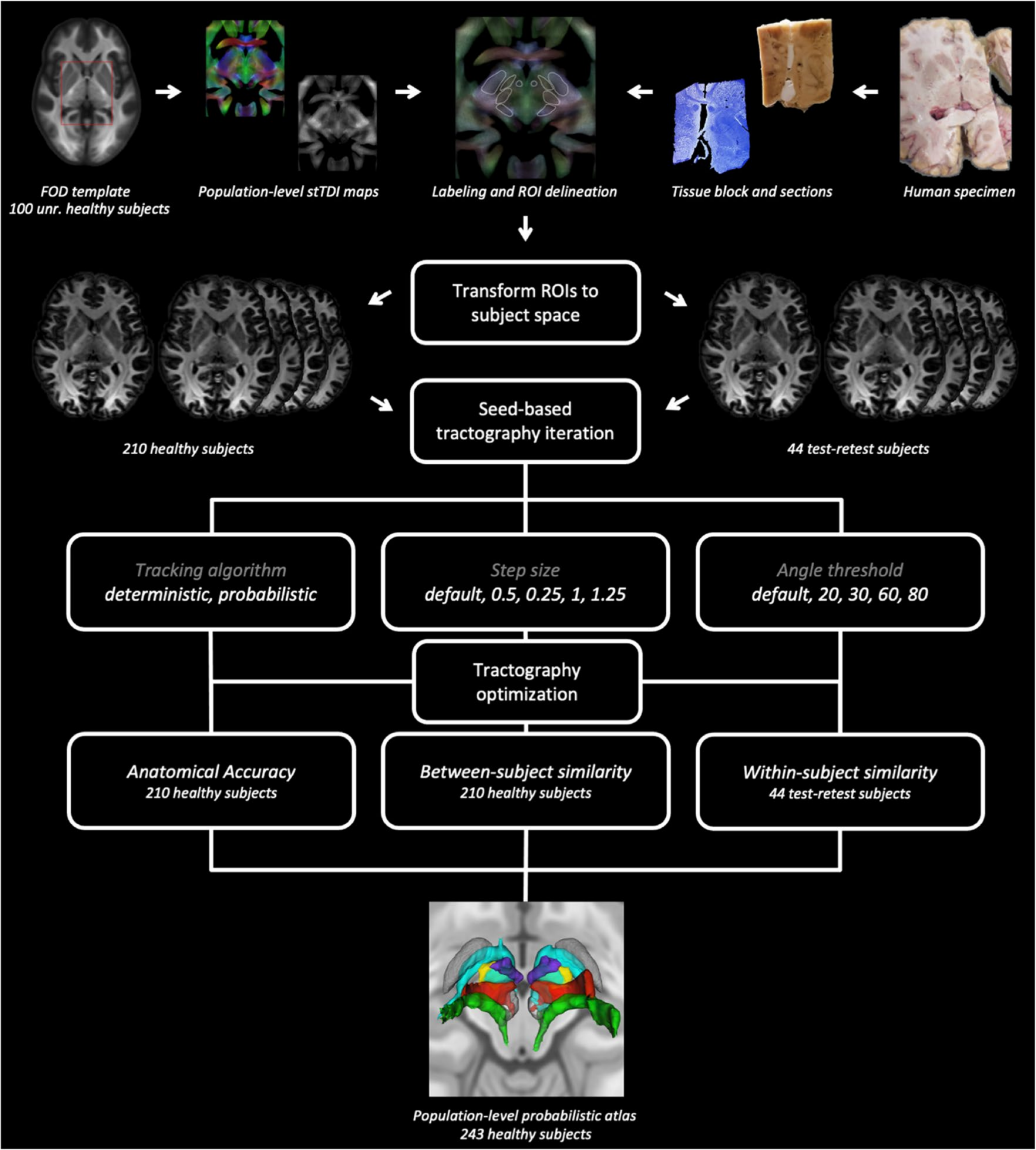
The processing pipeline in brief. The flowchart summarizes the processing and optimization pipeline that was devised to generate the population-level probabilistic atlas. The tractography optimization step tested the effects of different combinations of step size, tracking algorithm, and angle threshold on measures of within-subject, between-subject similarity, and anatomical accuracy. The overall best performing combination of parameters for each tract was selected for atlas generation. A detailed description of the procedure can be found in the main text

To select optimized parameters, the obtained outcome measures were averaged between left and right hemisphere and an overall average score of the three measures for each tract was obtained. The combination of parameters that maximized this overall similarity score (from now on referred to as "best working approach”) was selected for atlas generation. To generate the final atlas, binarized, template-space tract maps obtained from the best working approach were registered to MNI ICBM 2009 standard space (Fonov et al. 2009). We coregistered FOD template to standard space using a generic affine transformation by concatenating center-of-mass alignment, rigid, similarity, and fully affine transformations, followed by a nonlinear transformation (same parameters as for the individual FOD to FOD template registration, except for the similarity metric of the nonlinear transformation: mutual information) using the ANTs software, as detailed in our previous work (Basile et al. 2021a). The resulting direct transformation was applied to template-space binarized tract maps; then, standard space tract maps were summed up to obtain group-level tract maximum probability maps (MPMs). For each tract, MPMs were z-scored by subtracting each voxel’s intensity to the mean and dividing by the standard deviation. The resulting population atlas was obtained from a total of 243 subjects (i.e., the main dataset encompassing 210 subjects plus the 33 test scans of subjects of the test–retest dataset that were not part of the main dataset itself). Figure 1 summarizes the whole processing pipeline.

## Results

### Anatomy and delineation of tracts of interest on stTDI maps

**Fig. 2:**
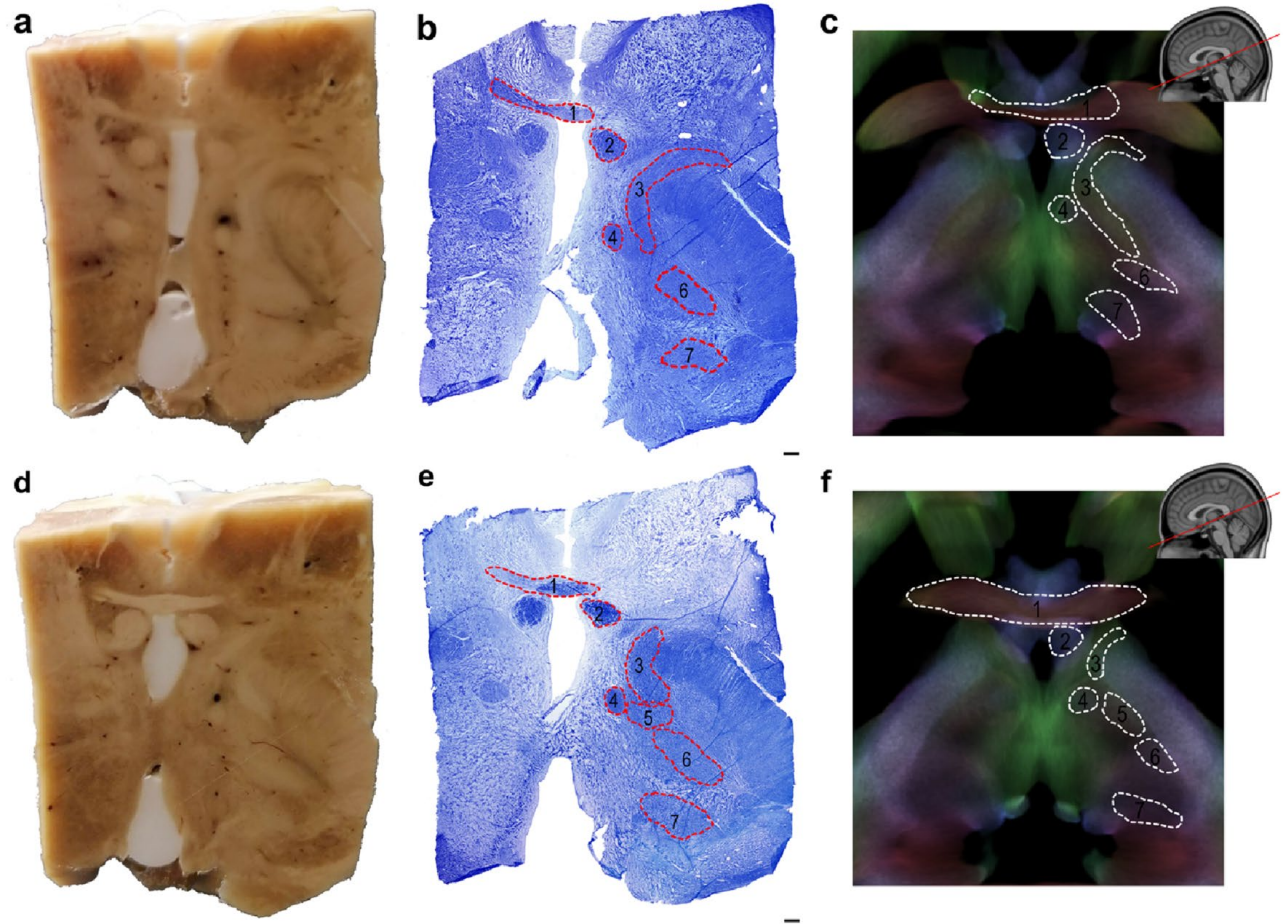
Anatomy of the human subthalamic region. **a**, **d** Macrophotographs of the human autoptic subthalamic area from an adult female of 60 years (case 7). **b**, **e** histological section of the brain tissue block shown in a and d, respectively, stained with Kluver and Barrera. **c**, **f** oblique axial sections of the template-level stTDI maps showing boundaries of the tracts of interest similar to those drawn in b and b, respectively. 1: anterior commissure; 2: column of the fornix; 3: ansa lenticularis; 4: mammillothalamic tract; 5: fasciculus lenticularis; 6: cerebello-thalamic tract; 7: medial lemniscus. Scale bars: b = a = e = d = 0.5 mm

All tracts of interest were successfully identified by visual comparison of histological sections from human specimens and stTDI maps (Fig. 2) and followed in ascending and descending axial sections for the whole length of their subthalamic area course.

**Fig. 3:**
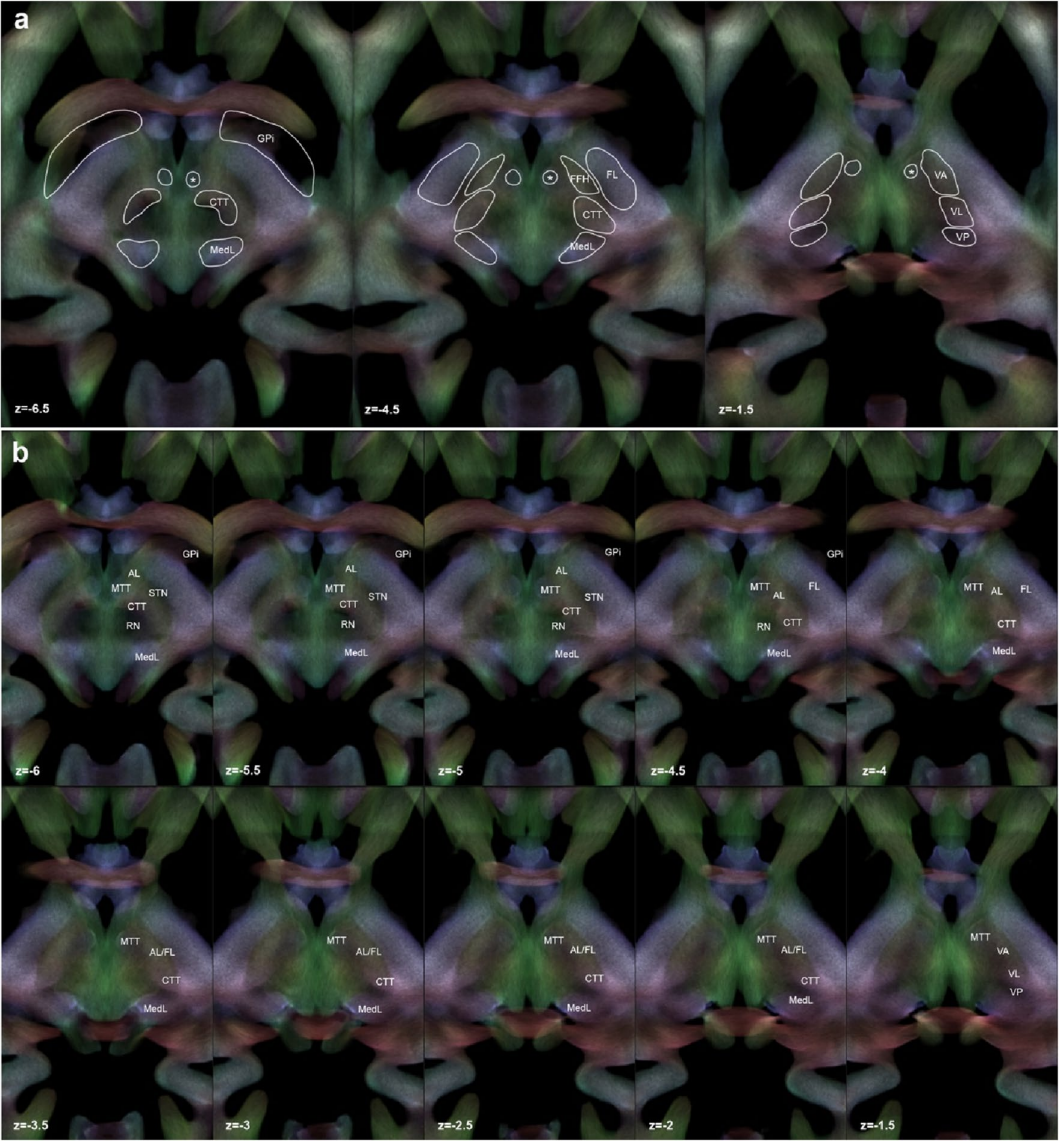
**a** Regions of interest (ROIs) delineation on template-level stTDI maps. The boundaries of the waypoint ROIs are shown on axial sections of the template-level stTDI maps. DEC-stTDI is shown with AFD-stTDI as overlay (~ 80% opacity). **b** Labeling and identification of white matter tracts of interest in the subthalamic area. Structures are labeled on ascending axial sections (slice increment: 0.5 mm) of template-level stTDI maps. DEC-stTDI are shown with AFD-stTDI as overlay (~ 80% opacity). *AL* ansa lenticularis, *CTT* cerebello-thalamic tract, *FL* fasciculus lenticularis, *FFH* Forel’s Field H, *Fx* fornix, *MTT* mammillothalamic tract, *MedL* medial lemniscus, *RN* red nucleus, *STN* subthalamic nucleus, *GPi* internal globus pallidus, *VA* ventral anterior thalamic nucleus, *VL* ventral lateral thalamic nucleus, *VP* ventral posterior thalamic nucleus, the asterisk (*) marks the mammillothalamic tract

The waypoint ROIs placed along the course of these tracts, as well as their mesodiencephalic course are shown in Fig. 3a, b.

### Pallidothalamic tracts (ansa lenticularis and fasciculus lenticularis)

The ansa lenticularis (AL) emerges at the anterior pole of GPi; its first segment is directed medially as it loops around the internal capsule and bends posteriorly to enter the subthalamic region, anteriorly to the STN, in the anterior portion of the prerubral field (Forel’s H); then it turns upwards to reach the thalamus (mainly VA thalamic nucleus) as part of the thalamic fasciculus (FT, Forel’s H1) (Chung and Park 2020; Holanda et al. 2020; Oishi et al. 2020; Middlebrooks et al. 2020). In axial stTDI maps, this tract is visible as it emerges from the GPi (hypointense region); in its first segment is mainly directed antero-posteriorly resulting in a green color on DEC maps, then it can be followed as a yellow–brown spot in ascending slices until it reaches the ventral thalamus. The first portion was manually segmented on TDI maps starting from the anterior-medial border of the GPi and using the internal capsule as lateral boundary, the fornix and the MTT as medial boundary, and the CTT as posterior boundary; ascending in axial sections, it was labeled maintaining the internal capsule as medial boundary, the MTT as anterior-medial boundary and the CTT as posterior-medial boundary. To guide tractography at the subject-level, the first ROI encompassing the GPi was traced at z = − 5.5; the second ROI was drawn in the anterior part of the prerubral field (Forel’s H) at z = − 4.5; finally, the third ROI was placed at upper level, precisely at the thalamic entry point corresponding to the VA nucleus (z = − 1.5). The fasciculus lenticularis (FL) also originates from the GPi but arises from its dorsomedial aspect and traverses the internal capsule perpendicular to the course of the cortico-spinal tract, forming a comb-like structure (Edinger’s comb system) (Middlebrooks et al. 2020); in the subthalamic region, it travels above the STN and below the zona incerta (Forel’s H2 field), then it joins the AL in Forel’s H field (Chung and Park 2020; Oishi et al. 2020), and finally reaches VA thalamus as part of the FT (Forel’s H1). While its emergence at the pallidal border was not readily identifiable in stTDI maps, the Edinger’s comb system was visible as a slightly hypointense region in the context of internal capsule (especially on AFD-stTDI maps); this feature may be partially explained by the presence of crossing fibers, decreasing the AFD values. To dissociate the course of FL from pallidosubthalamic fibers, that also travel the internal capsule as part of the Edinger’s comb system, FL was labeled starting from the superior pole of the STN. A first ROI was placed at this level in the hypointense region within the internal capsule (z = − 4.5); then, since the course of FL from Forel’s H to VA is not further dissociable from AL, the same ROIs used for AL tractographic reconstruction were employed as second and third waypoint.

### Cerebello-thalamic tract

The cerebello-thalamic tract (CTT) emerges from the deep cerebellar nuclei, exits the cerebellum through the superior cerebellar peduncle and partially decussates at the mesencephalic level (Meola et al. 2016a; Middlebrooks et al. 2020), travels through the red nucleus (RN) emerging from its anterior pole (Gallay et al. 2008; Basile et al. 2021b) and it finally traverses the Forel’s fields H and H1 to reach the ventral tier of thalamic nuclei, mainly the VL (Gallay et al. 2008; Kultas-Ilinsky et al. 2011). While the cerebellar origin of CTT was out of the stTDI bounding box, the course of CTT was labeled starting from the superior cerebellar peduncle and followed in ascending slices up to the midbrain decussation; after the decussation it crosses the RN (hypointense) and emerges in proximity of the anterior pole of the RN; here, the tract ascends in medio-lateral direction (purple color in the DEC-stTDI) and can be followed in ascending slices as it traverses Forel’s H field antero-laterally to the RN, until it reaches the ventral thalamus. For manual segmentation, in the caudal sections, the STN was employed as antero-lateral boundary, the RN as posterior-medial boundary and the AL as anterior boundary; ascending in axial slices, the CTT was bordered laterally by the internal capsule, anteriorly by the AL/FL region and posteriorly by the MedL. The first ROI was placed at z = − 5.5 at the emergence from the RN anterior pole; the second ROI was drawn at Forel’s H field posteriorly to AL/FL second ROI (z = − 4.5) and the third ROI, placed at the ventral thalamic level, roughly corresponds to the CTT entry zone into the VL (z = − 1.5).

### Mammillothalamic tract

The mammillothalamic tract (MTT) emerges from the anteromedial mammillary body behind the insertion of the fornix and travels the midbrain and subthalamic region to reach the ventral thalamus, then it penetrates the VA nucleus and finally terminates in the anterior thalamic nuclear complex (ANT) (Chung and Park 2020; Middlebrooks et al. 2020); in the subthalamic region, it marks the medial boundary of Forel’s H and H1 fields (Oishi et al. 2020). In ascending axial sections, it is easily recognizable as a round structure of light blue color (as being composed of mainly inferior-superiorly directed fibers); it remains well visible even in the context of the VA nucleus of the thalamus. Manual delineation started from z = − 6 where it is bordered medially by the hypothalamus and laterally by the AL; in the upper sections, it is surrounded by the hypointense VA nucleus. To guide tractography, three round ROIs were placed at z = − 5.5, z = − 4.5 and z = − 1.5.

### Medial lemniscus and ascending somatosensory pathway

The medial lemniscus (MedL) originates from the gracile and cuneate nuclei in the medulla oblongata; at the level of the midbrain it is part of a thick bundle of white matter including the trigeminal lemniscus and the spinothalamic tract, which are also involved in somatic sensation and reach together the VP thalamic nucleus (Neudorfer and Maarouf 2018). In the midbrain, it courses postero-laterally to the RN and in the subthalamic region, marking the posterior border of Forel’s H and H1 fields (Oishi et al. 2020). In DEC-stTDI maps it can be identified as a blue spot starting from the ponto-mesencephalic junction and ascending on route to the VP thalamus. It was manually segmented from z = − 6 using the RN as the anterior boundary in the lower sections and the CTT as the anterior boundary in the upper sections. The first ROI (z = − 5.5) was placed posterior to the RN; the second ROI was placed along its course at the posterior boundary of the prerubral fields; the third ROI was drawn at its entry point to VP thalamic nuclei.

### Effects of tracking algorithms and parameters on tracts reconstruction

**Fig. 4:**
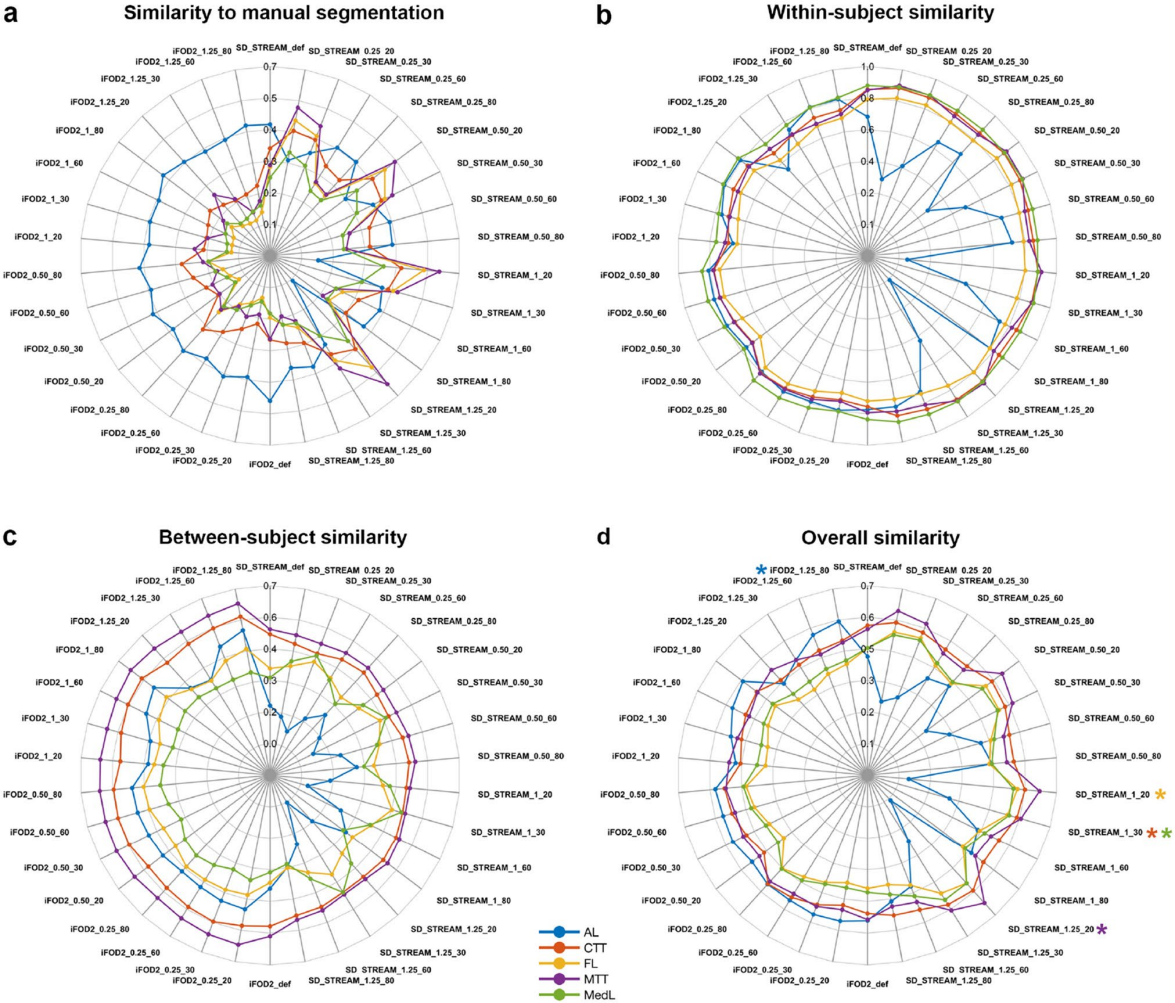
Results of tractography optimization. Spider plots show average values over all subjects of the similarity measures employed for tractography optimization, for each different combination of tracking parameters. Colored asterisks highlight the best working approach for each tract as emerged from the overall similarity analysis. *AL* ansa lenticularis, *CTT* cerebello-thalamic tract, *FL* fasciculus lenticularis, *MTT* mammillothalamic tract, *MedL* medial lemniscus

Figure 4 summarizes the results of tractography optimization in terms of similarity to manual segmentation, within-subject, and between-subject similarities, as well as the overall average similarity measure that was chosen to select the best working approach for atlas generation.

### Similarity to manual segmentation (anatomical accuracy)

For all the tracts of interest except for the AL, similarity to manual segmentation is generally higher with the deterministic fiber tracking algorithm (SD_STREAM) compared to the probabilistic algorithm (IFod2). For deterministic tracking, a similar trend can be observed, so that higher similarity is obtained with higher step sizes, and it decreases while increasing the angle threshold. The opposite trend can be observed for probabilistic tractography, where similarity appears to decrease slightly while increasing the step size, and to increase with higher angle thresholds. Notice that the AL constitutes a major exception, as it shows higher values with the probabilistic tracking algorithm, regardless of step size and angle threshold; when reconstructed with deterministic algorithms, the similarity to manual delineation increases with higher angle thresholds and decreases with larger step sizes.

### Within-subject similarity

For all the tracts of interest, again with the AL as the main exception, the test–retest similarity shows little differences in relation to tracking algorithms and parameters; however, slightly higher values are found using the deterministic algorithm, and regardless of the fiber tracking parameters employed; when using probabilistic algorithms, a slight increase in the within-subject similarity can be observed with smaller step sizes and higher angle thresholds. Again, AL is an exception as it shows remarkably lower within-subject similarity with the deterministic algorithm, and higher values are obtained using larger step sizes in combination with higher angle threshold.

### Between-subject similarity

For most of the tracts of interest, the between-subject similarity is higher with the probabilistic tracking algorithm. In general, CTT and MTT show relatively little differences related to tracking parameters and minimal differences related to the tracking algorithm of choice; by contrast, the AL shows marked differences between deterministic and probabilistic tracking and between-subjects similarity values are higher when using larger step sizes and higher angle threshold, combined with probabilistic tractography. The between-subject similarity of FL reconstruction is likely to be less influenced by the tracking algorithm of choice; with deterministic algorithms, it increases while increasing the step size, in particular when combined with an angle threshold of 30°, while with probabilistic algorithm values are higher with combinations of large step sizes and high angle threshold. Finally, the between-subject similarity of MedL is higher when using deterministic tracking algorithms, especially in combination with an angle threshold of 30° and larger step sizes.

### Best working tractography approach for atlas generation

Table 2 summarizes, for each tract of interest, the results in terms of anatomical course, ROI placement and tractography approach that was chosen for atlas generation.

**Table 2:** Anatomical course, ROI placement, tractography approach chosen for atlas generation, and clinical relevance of each tract of interest

Tract name	Anatomical course	ROI placement	“Best working” pipeline	Clinical relevance
Ansa lenticularis (AL)	GPi to VA; it emerges at the anterior pole of GPi, travels anteriorly to the internal capsule, bends posteriorly to join the FL in Forel’s field H, then it reaches VA through the thalamic fasciculus (FT)	ROI 1: GPi; ROI 2: Forel’s Field H; ROI 3: VA	Probabilistic tractography; step size: 1.25 mm; angle threshold: 80°	Dystonia, parkinsonism, dyskinesia
Fasciculus lenticularis (FL)	GPi to VA: it emerges at the medial border of GPi, crosses the internal capsule forming the Edinger’s comb system, joins the AL in Forel’s field H, then it reaches VA through the thalamic fasciculus (FT)	ROI 1: internal capsule (Edinger’s comb system); ROI 2: Forel’s Field H; ROI 3: VA	Deterministic tractography; step size: 1 mm; angle threshold: 20°	Dystonia, parkinsonism, dyskinesia
Cerebello-thalamic tract (CTT)	DN to VL: it emerges at the hilum of dentate nucleus, exits the cerebellum through the superior cerebellar peduncle, partially decussates in the midbrain, courses through the red nucleus, then reaches VL through the thalamic fasciculus (FT)	ROI 1: anterior pole of the red nucleus; ROI 2: Forel’s Field H (postero-lateral to AL/FL ROI); ROI3: VL	Deterministic tractography; step size: 1 mm; angle threshold: 30°	Essential tremor, tremor-dominant Parkinson’s Disease
Mammillothalamic tract (MTT)	MB to Ant: it emerges from the anteromedial mammillary bodies anterior to the fornix, traverses shortly the midbrain and courses through the VA nucleus of the thalamus, then reaches Ant	ROI 1: MTT course in the inferior midbrain; ROI 2: MTT course in the upper midbrain; ROI3: MTT course through VA nucleus	Deterministic tractography; step size: 1.25 mm; angle threshold: 20°	Epilepsy
Medial lemniscus (MedL)	Gracile and cuneate nuclei to VP: it emerges from gracile and cuneate nuclei in the medulla, crosses the midline at the ponto-medullary junction and courses medially through the pons; in the midbrain, it courses in medio-lateral direction and travels behind the red nucleus, then reaches VP	ROI 1: MedL course posterior to the red nucleus ROI 2: MedL course posterolateral to the red nucleus and CTT ROI3: VP	Deterministic tractography; step size: 1 mm; angle threshold: 30°	Unwanted side effects of DBS (e.g., paresthesia)

*AL* ansa lenticularis, *FL* fasciculus lenticularis, *CTT* cerebello-thalamic tract, *MedL* medial lemniscus, *FT* thalamic fasciculus, *GPi* internal segment of the globus pallidus, *VA* ventral anterior thalamic nucleus, *VL* ventral lateral thalamic nucleus, *MB* mammillary body, *Ant* anterior nuclear group, *VP* ventral posterior thalamic nucleus, *DBS* deep brain stimulation

**Fig. 5:**
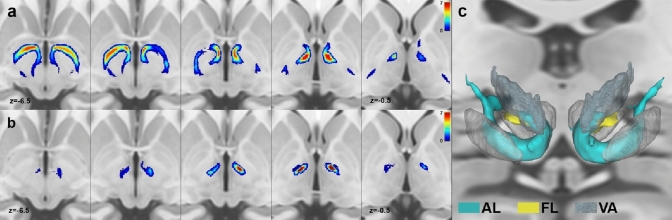
Pallidothalamic tracts. Maximum probability maps are z-scored and overlaid on ascending axial sections (slice increment: 1.5 mm) of the T1 MNI ICBM 2009b standard template. **a** Ansa lenticularis; **b** fasciculus lenticularis; **c** 3D anatomical reconstruction of the pallidothalamic tracts; red nucleus, subthalamic nucleus and internal globus pallidus, as well as the ventral anterior thalamic nucleus (light blue, transparent) are shown for reference. Renderings are based on a 3D histological atlas of the human thalamus (Ilinsky et al. 2018)

**Fig. 6:**
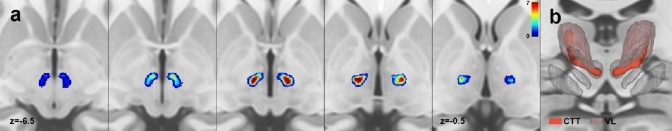
Cerebello-thalamic tract. **a** Maximum probability maps of left and right cerebello-thalamic tract are z-scored and overlaid on ascending axial sections (slice increment: 1.5 mm) of the T1 MNI ICBM 2009b standard template. **b** 3D anatomical reconstruction of the cerebello-thalamic tract; red nucleus, subthalamic nucleus and internal globus pallidus, as well as the ventral lateral thalamic nucleus (red, transparent) are shown for reference. Renderings are based on a 3D histological atlas of the human thalamus (Ilinsky et al. 2018)

**Fig. 7:**
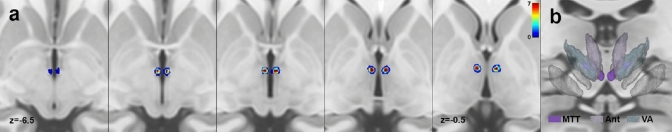
Mammillothalamic tract. **a** Maximum probability maps of left and right mammillothalamic tracts are z-scored and overlaid on ascending axial sections (slice increment: 1.5 mm) of the T1 MNI ICBM 2009b standard template. **b** 3D anatomical reconstruction of the mammillothalamic tract; red nucleus, subthalamic nucleus and internal globus pallidus, as well as the ventral anterior thalamic nucleus (light blue, transparent) and anterior nuclear group (purple, transparent) are shown for reference. Renderings are based on a 3D histological atlas of the human thalamus (Ilinsky et al. 2018)

**Fig. 8:**
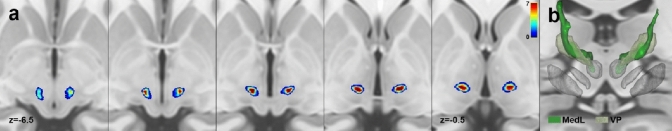
Medial lemniscus. **a** Maximum probability maps of left and right medial lemniscus are z-scored and overlaid on ascending axial sections (slice increment: 1.5 mm) of the T1 MNI ICBM 2009b standard template. **b** 3D anatomical reconstruction of the medial lemniscus, red nucleus, subthalamic nucleus and internal globus pallidus, as well as the ventral posterior thalamic nucleus (green, transparent) are shown for reference. Renderings are based on a 3D histological atlas of the human thalamus (Ilinsky et al. 2018)

For all the tracts of interest except for the AL, deterministic tractography in combination with larger step sizes (1 or 1.25 mm) and medium–low angle threshold (between 20° and 30°) outperforms probabilistic tractography; for AL, by contrast, the best overall results were obtained using the probabilistic algorithm combined with maximal step size (1.25) and angle threshold (80°). MPMs and 3D reconstruction for each tract, obtained with the best tractography pipelines as emerged from tractography optimization are displayed in Figs. 5, 6, 7 and 8.

## Discussion

This study provides for the first time a large-scale, in vivo probabilistic reconstruction of clinically and surgically relevant fiber bundles that traverse the subthalamic region en route to the thalamus. Disentangling the high anatomical complexity of the subthalamic region has been challenging for conventional imaging and tractography in the past years. Even at the highest resolution available and with dedicated acquisition sequences, structures of the subthalamic region are difficult to visualize on an individual basis using morphological MRI scans (Lau et al. 2020). In the current study, we leveraged the high spatial and angular resolution DWI data provided by the HCP repository (Uǧurbil et al. 2013) and devised a dedicated protocol for the identification and virtual dissection of subthalamic white matter structures, by employing a group-level template based on TDI (Calamante et al. 2012b) and directly comparing it with histological sections of a human specimen. TDI is a technique based on tractography data allowing streamlines to be mapped on a given grid element (Calamante et al. 2010). TDI assigns to each voxel an intensity that is proportional to the number of streamlines, either by itself or after attributing to each streamline a weight based on other imaging-derived measures (Calamante 2017). Such an approach permits obtaining images characterized by high anatomical contrast between grey and white matter as well as super-resolution properties (Calamante et al. 2011). Studies comparing super-resolution TDI maps with post-mortem histology in non-human and human brains have demonstrated that TDI maps can be effectively compared to histological images to guide the identification of white matter and gray matter structures (Calamante et al. 2012a, 2013; Dai et al. 2018; Basile et al. 2021a; Kwon et al. 2021). Herein, we showed how the complementary information about fiber orientation and fiber density, as retrieved by DEC-stTDI and AFD-stTDI, respectively, made possible the identification, labeling, and delineation of the white matter structures of interest along their course throughout the subthalamic region. In particular, differences in tract orientation allowed dissociating pallidothalamic and cerebello-thalamic tracts, which course the Forel’s H and H1 fields very close to one another (Gallay et al. 2008). In color-coded stTDI axial sections, the pallidothalamic connections are colored in yellow–brown due to their ascending antero-posterior and medio-lateral orientation, while cerebello-thalamic fibers, which are directed medio-laterally on their course to the ventral thalamus, are visible in purple color. Fiber density information, by contrast, was particularly useful to localize the crossing point of the FL through the internal capsule. In this portion, the FL consists of thin, interdigitated white matter tracts that cross the internal capsule at approximately 90 degrees forming a comb-like structure (Edinger’s comb system) (Middlebrooks et al. 2020). While not directly reconstructed by short tracking, this structure was indirectly localized as a hypointense region circumscribed to the internal capsule, extending from the level of the superior margin of STN (Fig. 3). Taken together, our results confirm the usefulness of TDI methods in providing relevant insight on brain white matter anatomy in vivo.

While possible, at the individual level, manual segmentation based on stTDI is time consuming and requires prior knowledge and expertise on the anatomy of the subthalamic region. Hence, a ROI-driven tractography approach was performed to segment tracts of interest at the subject level, after the non-linear transformation of group-level ROIs to each subject’s space.

While, in the last decades, tractography has confirmed its potential as a valuable tool to reconstruct white matter anatomy in vivo in the human brain (“virtual dissection”) (Fernández-Miranda et al. 2015; Hau et al. 2016, 2017; Sarubbo et al. 2016; Cacciola et al. 2019; Maffei et al. 2019; Bertino et al. 2020a, b), its direct application to tracts crossing the subthalamic region has been hampered by intrinsic methodological limitations. Indeed, the subthalamic region is densely packed with multiple independent white matter structures coursing together through a small, “bottleneck” region (the Forel’s fields) (Chung and Park 2020) which is challenging to investigate at the resolution of clinically employed DWI volumes (around 2 mm^3^ voxel size); in addition, tracking algorithms often struggle in disentangling complex fibers configuration such as kinking, crossing and fanning fibers and are sensitive to intra-voxel orientation heterogeneity (Girard et al. 2014; Knösche et al. 2015; Schilling et al. 2018). It is probably for these reasons that small and tortuous fiber structures such as the pallidothalamic tracts (ansa lenticularis and fasciculus lenticularis) are often lacking in large-scale cortical and brainstem white matter atlases (Meola et al. 2016b; Tang et al. 2018; Yeh et al. 2018).

It is well known that tractography performance in regions characterized by complex fiber configurations is strongly influenced by the selection of tracking parameters such as deterministic or probabilistic tracking, step size, or angle threshold (Girard et al. 2014; Maier-Hein et al. 2017; Maffei et al. 2019). According to the sampling of fiber directions for streamline propagation, tracking algorithms can be categorized into deterministic and probabilistic. The former is based on the estimation of the main diffusion direction, the streamlines being steered according to a fixed direction at each voxel. The probabilistic tractography instead estimates a probability distribution of fiber orientations for each voxel, thus allowing streamline tracking also through areas of high orientational uncertainty (Descoteaux et al. 2009; Jones 2010). It is still a matter of debate if deterministic tractography is superior to probabilistic tracking in terms of anatomical accuracy of the reconstructed course of white matter pathways. A finding often reported in the field is that deterministic tractography tends to reconstruct fewer incorrect connections (false positives), at the cost of a less complete reconstruction of the course of white matter bundles (sensitivity), compared to probabilistic tracking algorithms (Thomas et al. 2014; Schilling et al. 2019). However, most of the existing papers compared deterministic and probabilistic tractography with their respective default parameters and did not account for the impact of different parameters on this tradeoff (Grisot et al. 2021).

In the present work, we thoroughly investigated the effects of tracking parameters both in terms of within- and between-subject similarities and adherence to a priori anatomy derived from the template-level manual delineation. Our results confirm that each parameter combination may have different impacts both on subject-level and group-level similarity and in terms of correspondence to template-level anatomical priors, thus highlighting the need for selecting a proper trade-off between anatomical accuracy and stability of tractography algorithms (Schilling et al. 2018; Grisot et al. 2021).

Interestingly, our findings also demonstrate that the choice of optimal tractography parameters may vary for each tract, according to the anatomical features of the tract of interest. As an example, the AL, which is characterized by a complex course with a high degree of curvature, was reconstructed with higher anatomical accuracy using probabilistic tractography and relatively permissive angle thresholds (such as 60° or 80°) and resulted in higher within-subject and between-subject similarity; by contrast, tracts with an almost straight course, such as the MTT, were reconstructed with higher anatomical accuracy by employing deterministic tractography and stricter angle thresholds. Taken together, our results suggest that, when tractography is employed to investigate the anatomy of a given white matter structure, a “one fits all” approach may not be recommended, and, by contrast, a careful personalization of tracking parameters based on the geometry of the pathway of interest may lead to more accurate and reproducible results.

The reconstructions provided in our optimized, group-level atlas may be useful to add more insight into the three-dimensional anatomy of clinically and surgically relevant fiber tracts in the human brain in vivo. To date, the most complete reconstructions of the spatial configuration of such structures are based on ex vivo tractography with ultra-high-field MRI (Plantinga et al. 2016; Oishi et al. 2020).

The few works attempting in vivo reconstruction of the pallidothalamic tracts, such as the AL or FL, are often based on small clinical samples (Rozanski et al. 2017; Schlaier et al. 2017) or group-averaged DWI datasets (Middlebrooks et al. 2020; Li et al. 2021). During its course from the anteromedial GPi, the AL first loops around the internal capsule describing a curve with anterior concavity and then it bends upwards forming a second curve at the level of the tegmentum to reach the ventral thalamus (Chung and Park 2020). While available tractographic reconstructions often struggle with reconstructing the second curve of the AL and sometimes miss the thalamic entry point at the level of the VA nucleus, this portion of the tract is successfully reconstructed in our in vivo probabilistic atlas. In addition, while the central portion of the FL, which traverses the internal capsule almost perpendicular to the pyramidal tract, was partially reconstructed in many subjects, and consequently not visualized on the group-level images, our probabilistic maps were able to show the tegmental portion of the FL, which passes above the STN, and its thalamic entry point.

Many existing works employed tractography to reconstruct the CTT, both in healthy subjects or clinical cohorts (Granziera et al. 2009; Kwon et al. 2011; Palesi et al. 2015; Schlaier et al. 2017; Tang et al. 2018; Nowacki et al. 2019; Bertino et al. 2021). However, most of them reconstruct this tract including also its thalamo-cortical and cerebello-thalamic connectivity. In line with post-mortem investigations and imaging (Gallay et al. 2008; Lau et al. 2020), in our group level atlas, the CTT leaves the anterior pole of the red nucleus describing an almost 90° angle respect to the sagittal plane, during its course in the posterior subthalamic region.

To our knowledge, studies employing tractography to reconstruct the MTT are sparse (Kwon et al. 2010; Kamali et al. 2018). Despite the lack of the emergence at the level of the mammillary bodies, our group-level reconstruction captured the slight curvature and lateral convexity of the MTT during its course as the medial border of Forel’s fields H and H1 and follows the course of the tract through the VA nucleus of the thalamus (Chung and Park 2020). Finally, our atlas provides a reconstruction of the mesencephalic course of ascending somatosensory pathways, such as the medial and trigeminal lemniscus, which border the subthalamic region as part of a joint fiber bundle (lemniscal radiations) *en route* to the VP thalamic nucleus; while being already present in large-scale population tract atlases (Meola et al. 2016b; Tang et al. 2018; Yeh et al. 2018), we opted to include this bundle in our atlas as it constitutes the posterior boundary of Forel’s fields H and H1.

A probabilistic, population atlas of white matter structures of the subthalamic region has potential clinical relevance, in particular for stereotactic neurosurgery. The advent of MRI-guided focused ultrasound (FUS), which allows for minimally invasive ablative surgery in patients with treatment-resistant movement disorders (Bond et al. 2017; Fishman and Frenkel 2017; Martínez-Fernández et al. 2018; Gallay et al. 2019), has renewed the interest towards focal interventions such as subthalamotomy, pallidotomy or thalamotomy. Information on the anatomy of white matter structures which are involved in the ablation region may be helpful for appropriate target definition as well as for pre-surgical targeting, to improve treatment outcomes and reduce unwanted side effects. As an example, it has been suggested that enlarging the ablation target to include pallidothalamic fibers may help reduce the incidence of treatment-induced hemichorea or ballism in FUS subthalamotomy (Máñez-Miró et al. 2021). In addition, advances in structural neuroimaging have allowed investigating target connectivity in functional neurosurgery, leading to a redefinition of traditional targets in light of the emerging concept of “connectomic neuromodulation” (Li et al. 2020; Horn and Fox 2020). The pallidothalamic and cerebello-thalamic tracts are long-time known to mediate the beneficial effects of functional neurosurgery in movement disorders (Gallay et al. 2008). Confirming such a hypothesis, it has been demonstrated that the anti-tremorgenic effects of Vim-DBS are higher when stimulation is derived in the proximity of the CTT (Al-Fatly et al. 2019), while the involvement of the pallidofugal tracts was related to improvements in dyskinesia (Aquino et al. 2019). In this scenario, our population-based, probabilistic template of the white matter of the human subthalamic area could be helpful to elucidate the effects of invasive neuromodulation techniques and improving correlation between sites of stimulation and clinical outcomes.

There are several limitations that apply to the present study. First, despite optimization of tractography, the tracts obtained by our best working parameters may be affected by false positive streamlines. Consequently, the anatomical accuracy of the tracts of interest remained inherently low even for the highest scoring parameter combinations. This may be partly explained by the inherently conservative nature of the manual tract delineations made on stTDI maps aided by identification of white matter and gray matter structures on histological sections of human specimens; in addition, comparison to the manual segmentation was not individualized, but rather performed according to a group-level estimate manually drawn in template space. While most of the spurious fiber tracts are also characterized by low between-subject consistency (Roberts et al. 2017) and are subsequently filtered out after group aggregation of individual tractograms (especially after z-thresholding of the resulting maximum probability maps), it is worth noting that even at the group-level and after optimization of tracking parameters, the reconstruction of short-range white matter pathways in regions of crossing fibers and intra-voxel heterogeneity may still incorporate false-positive results.

Another relevant limitation is due to the choice of outcome measures that were selected for tractography optimization. Herein, we employ both measures of test–retest similarity, between-subject consistency, and adherence to group-level delineation; when choosing an appropriate parameter combination, we considered all these measures of equal importance by computing an overall mean. However, it should be kept in mind that a different best working combination of tracking parameter can be identified for every outcome measure. Finally, our work leverages the high spatial and angular resolution provided by the HCP acquisition protocol. While we provided information on how diffusion parameters impact the reliability of tractography on high-quality data, caution is required in generalizing our results to single-shell, lower b-value or lower spatial and angular resolution data, such as those employed in clinical settings. While it may be assumed that accurate reconstruction of white matter small tracts in crossing fibers regions would be even more challenging in low quality datasets, further investigations are warranted to identify optimized tractography parameters that may work also on real-world DWI acquisitions.

In conclusion, we devised a histological aided protocol for visualization and identification of white matter tracts of the human subthalamic area based on stTDI. We demonstrated that the complex anatomy of this region can be disentangled in vivo using tailored regions of interest and dedicated tractography pipelines. Finally, we provide the first population-level probabilistic atlas of the human subthalamic white matter bundles. We hope it could represent a useful tool to further improve our understanding of complex white matter structures and their implications in the pathophysiology and treatment of different brain disorders.

## Data Availability

MRI data were provided by the Human Connectome Project, WU‐Minn Consortium (Principal Investigators: David Van Essen and Kamil Ugurbil; 1U54MH091657) and are publicly available at https://www.humanconnectome.org/study/hcp-young-adult/document/1200-subjects-data-release. Codes and data generated for the present work are available at https://doi.org/10.5281/zenodo.6832797. The probabilistic atlas of the subthalamic area is also available at https://github.com/BrainMappingLab.
